# Combined use of stannous fluoride-containing mouth rinse and toothpaste prevents enamel erosion in vitro

**DOI:** 10.1007/s00784-023-05138-4

**Published:** 2023-07-11

**Authors:** Apichaya Jiemkim, Thipawan Tharapiwattananon, Siriporn Songsiripradubboon

**Affiliations:** Department of Pediatric Dentistry, Faculty of Dentistry, Chulalongkorn University, 34 Henri Dunant, Road, Pathumwan, Bangkok, 10330, Thailand

**Keywords:** Erosion, Enamel, Stannous fluoride, Toothpaste, Mouth rinse

## Abstract

**Objective:**

To compare the protective effect of commercial stannous-containing mouth rinses on enamel erosion in a simulated 5-day in vitro cycling model.

**Materials and methods:**

81 human enamel specimens were embedded in resin blocks and divided into nine groups as follows; group 1: stannous fluoride (1000SnF_2_) toothpaste; groups 2,3, and 4 were the same as group 1 plus Elmex®, PerioMed™, and Meridol®, respectively, group 5: stannous fluoride (1450SnF_2_) toothpaste, groups 6, 7, and 8 were the same as group 5 plus Elmex®, PerioMed™, and Meridol®, respectively, group 9: negative control. An erosive challenge was induced with a 1 min hydrochloric acid (0.01 M, pH 2.2) treatment 3 times per day. Each cycle included immersing in the toothpaste slurry twice for two minutes and a one-minute rinse. The enamel slabs were immersed in artificial saliva between each erosive cycle and incubated overnight at 37 °C. Surface hardness loss and enamel loss were determined by Knoop surface hardness and non-contact profilometry, respectively. Finally, enamel surfaces were analyzed by scanning electron microscopy and X-ray energy dispersive spectroscopy (SEM/EDS).

**Results:**

All three mouth rinses had similar protective effects against erosion when using adjunct with 1000 SnF_2_ toothpaste (*p* > 0.05). With 1450 SnF_2_ toothpaste, Elmex® presented significantly lower surface hardness loss than Meridol® (*p* < 0.05). The combined use of Elmex® or PerioMed™ with toothpaste provided significantly better erosion protection than toothpaste alone, either 1000 or 1450 SnF_2_. In addition, 1000SnF_2_ toothpaste adjunct with mouth rinse is comparable to 1450 SnF_2_ toothpaste alone in preventing enamel erosion.

**Conclusion:**

All three mouth rinses reduced enamel erosion. The additional use of a high concentration stannous containing mouth rinse with 1450 SnF_2_ toothpaste increases the protective effect against enamel erosion in vitro.

**Clinical significance:**

To date, no standard protocol for preventing dental erosion is available. There are three stannous-containing mouth rinses on the market; however, no study compared their efficacy or indicated whether using adjuncts with anti-erosion toothpaste provides additional benefits. This study found that adding stannous mouth rinse to twice-daily toothpaste increases erosion protection.

## Introduction

Dental erosion is an irreversible loss of tooth substance, which can be caused by a chemical reaction of nonbacterial acids. These acids may be of extrinsic or intrinsic origins [1]. The intrinsic cause is mainly gastric acid or hydrochloric acid, which is produced by the parietal cells in the stomach and has a pH of 1–1.5 [2, 3]. This acid may contact the teeth during vomiting, regurgitation, reflux, or psychosomatic disorder such as stress-induced vomiting, anorexia, and bulimia nervosa. Among intrinsic factors, gastroesophageal reflux disease (GERD) is the most common cause of dental erosion [4–8]. According to a systematic review in 2018, the prevalence of dental erosion ranged from 10.6% to 42%, with a median value of 25.5%. Individuals diagnosed with GERD had a higher mean prevalence of dental erosion, which was found to be 48.81%, compared to those without GERD [9].

When enamel erosion occurs at an early stage, the enamel will lose its mineral content, causing enamel softening [10]. The softened enamel, which has a lower resistance to physical force, is easily worn off by mechanical force. Consequently, loss of enamel structure will develop later in the erosion process [10–12]. Dental erosion may cause several clinical problems, such as dentin hypersensitivity and poor esthetic. A severe form of dental erosion can result in a shortening of teeth and a loss of vertical dimension of occlusion [6, 9]. Several factors are known to contribute to dental erosion, particularly certain eating and drinking habits [6, 13]. Excessive consumption of acidic foods or drinks, as well as holding or swishing citrus fruits or soft drinks in the mouth before swallowing, can increase the risk of dental erosion [14, 15]. The best way to prevent dental erosion is to minimize acid exposure. Thus, the main strategy for controlling and reducing dental erosion is educating individuals on healthy habits and raising awareness about modifying their diet or behaviors contributing to erosion [6, 13, 16]. Local preventive measures are also crucial, especially when dental erosion is severe and associated with a health issue that may require long-term medical treatment [6, 13, 15, 17, 18].

One of the strategies to prevent dental erosion is the use of topical fluorides in a form that can be applied by the patient themselves [17, 18]. Tooth brushing with fluoride-containing toothpaste twice daily is generally recommended as routine oral health care [19]. Additionally, mouth rinse is a common and easily accessible product that can be used daily for oral hygiene. For patients with more severe conditions, the use of mouth rinse can be proposed as an extra strategy to enhance the effectiveness of fluoride toothpaste in preventing dental erosion. Among the large number of commercially available fluoride-containing products, fluoride solution containing stannous has been considered the better option for controlling tooth erosion when compared to other fluoride solutions [12, 20–24]. In vitro studies have demonstrated the protective effect of SnF_2_ solutions against enamel erosion, either alone [15] or combined with other fluoride solutions such as AmF and NaF [23, 25]. The mechanism of action of stannous is based on the formation of a layer rich with acid resistant precipitates [26, 27].

There are now three commercially marketed mouth rinses that contain stannous: Elmex®, PerioMed™, and Meridol®. Stannous and fluoride are the two active components of the three mouth rinses. Despite having varying ion concentrations and Sn:F ratios, these mouth rinses have demonstrated protective effects against tooth erosion[20–22, 24, 25, 28–36]. Surprisingly, no research has been performed in which the effect of these three mouth rinses on human enamel erosion was compared. Therefore, the present study aimed to compare the effect of three marketed stannous-containing mouth rinses on the prevention of erosion when used in combination with stannous-containing toothpaste.

## Materials and methods

### Specimen preparation

The study protocol was approved by the Ethics Committee (Reference number HREC-DCU 2020–120) and the Institutional Biosafety Committee (DENT CU-IBC 009/2021), of the Faculty of Dentistry, Chulalongkorn University, Thailand. Human permanent molar teeth extracted following an individual treatment plan were used in this study. The extracted teeth were stored in 0.1% thymol solution before preparation. The buccal and/or lingual surfaces of the teeth were inspected with a stereomicroscope (SZ 61, OLYMPUS, Japan) at 30 × magnification. Teeth included in this study were free of caries, white spot lesions, hypoplasia, restorations, cracks, and other enamel defects. The natural surfaces were sequentially ground flat using a polishing machine (MINITECH 233, PRESI, France) with 600 and 800 silicon carbide abrasive paper until an experimental area of approximately 3 × 3 mm was achieved. A slow-speed cutting machine (ISOMET1000™, USA) was used to section the teeth into 3 × 3x2 mm blocks. These enamel samples were mounted in acrylic resin and polished with 1000 and 1200 silicon carbide abrasive papers and fine polished with aluminum oxide powder. Next, the enamel slabs were cleaned in an ultrasonic bath with deionized water (DI) for 3 min.

**Fig. 1:**
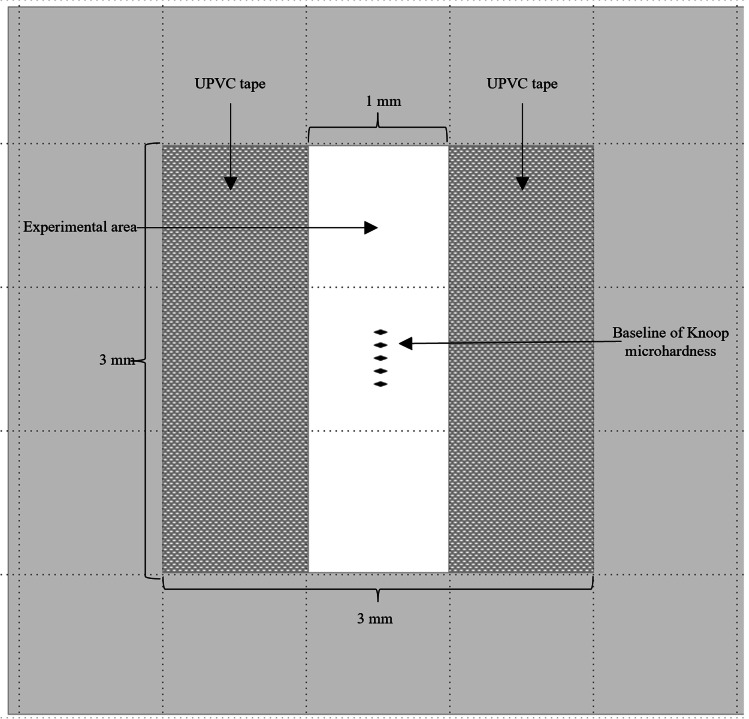
An illustration of the experimental area on the surface of the specimen

To serve as the reference area for profilometric measurement, the right and left outer parts of each specimen were covered with UPVC tape (Scotch® tape 600). The exposed area 1 × 3 mm in the center of each specimen was subjected to the treatment as shown in Fig. 1. Before starting scanning, the treated surfaces were checked under a microscope for possible tape leakage. If leakage was detected, the samples were excluded. Prior to the experiment, all specimens were examined for baseline surface hardness values by placing 5 indentations, 100 µm apart from each other, using a Knoop hardness tester with a load of 50 g and a dwell time of 5 s (FM-810, FUTURE-TECH, Japan) [37].

Eighty-one enamel specimens with a mean hardness of 305.22 ± 18.21 KHN were selected and assigned into 9 groups using block randomization: group 1 = 1000SnF_2_ toothpaste (1000SnF_2_) + deionized water (DI); group 2 = 1000SnF_2_ + Elmex®; group 3 = 1000SnF_2_ + PerioMed™; group 4 = 1000SnF_2_ + Meridol®; group 5 = 1450SnF_2_ toothpaste (1450SnF_2_) + DI; group 6 = 1450SnF_2_ + Elmex®; group 7 = 1450SnF_2_ + PerioMed™; group 8 = 1450SnF_2_ + Meridol®; and group 9 = non F toothpaste + DI (negative control). Details of all products are presented in Table 1.

**Table 1:** Descriptive of toothpaste and mouth rinse products tested

Product	Symbol	Ingredients	Active Agents	pH
Sensodyne rapid relief^a^	1450SnF_2_	Glycerin, PEG-8, Hydrated Silica, Pentasodium Triphosphate, Aroma, Sodium Lauryl Sulfate, Titanium Dioxide, Carbomer, Stannous Fluoride, Cocamidopropyl Betaine, Sodium Saccharin, Sodium Fluoride, Limonene	Stannous content: not declared Fluoride content: 1450 ppm F^−^ (1100 ppm as SnF_2_, 350 as NaF)	6.7^*^
Sensodyne rapid action^b^	1000SnF_2_	Glycerin, PEG-8, Hydrated Silica, Pentasodium Triphosphate, Aroma, Sodium Lauryl Sulfate, Titanium Dioxide, Carbomer, Stannous Fluoride, Cocamidopropyl Betaine, Sodium Saccharin, Limonene	Stannous content: not declared Fluoride content: F^−^ 1040 ppm F^−^ as SnF_2_	6.7^*^
Kindee Oral Gel Organic: Fluoride Free^c^	non F	Aqua, Sorbitol, Acrylate/C10-30 Alkyl Acrylate Crosspolymer, Xylitol, Propanediol, Cellulose Gum, Sodium Benzoate, Xanthan, Gum, Sodium Lauroyl Sarcosinate, Flavor, PEG-40, Hydrogenate Castor Oil, Sodium Saccharin, Potassium Sorbate, Disodium EDTA, Glycerin, Calcium Phosphoryl Oligosaccharides, Fragaria versa (Strawberry), Fruit Extract, Aloe Bardadensis (Aloe vera), Leaf Juice, Phenoxyethanol, CI 14700	-	6.4^*^
Elmex® EROSION PROTECTION dental rinse^d^	Elmex®	Aqua, Glycerin, Sodium Gluconate, PEG-40, Hydrogenated Castor Oil, Olaflur, Aroma, Stannous Chloride, Sodium Fluoride, Cocamidopropyl Betaine, Sodium Saccharin	Stannous content: 800 ppm Sn^2+^ as SnCl_2_ Fluoride content: 500 ppm F^−^ (125 ppm F^−^ as AmF, 375 ppm F^−^ as NaF)	4.2^**^
PerioMed™ 0.63% Stannous Fluoride Oral Rinse^e^	PerioMed™	Stannous Fluoride, Flavor, Glycerin, Methyl Paraben, Propyl Paraben, Sodium Saccharin	Stannous content: 750 ppm Sn^2+^ as SnF_2_ Fluoride content: 380 ppm F^−^ as SnF_2_	3.2^**^
Meridol® mouthwash^d^	Meridol®	Aqua, Xylitol, PVP, PEG-40 Hydrogenated, Castor Oil, Olaflur, Aroma, Stannous Fluoride, Sodium Saccharin, CI42051	Stannous content: 409 ppm Sn^2+^ as SnF_2_ Fluoride content: 250 ppm F^−^ (125 ppm F^−^ as AmF, 125 ppm F^−^ as SnF_2_)	3.5^**^

Superscript letters indicate manufacturer, city, and country:

^a^GlaxoSmithKline, Taiwan; ^b^ GlaxoSmithKline, Thailand; ^c^ Kindee Kiddee Kids Co., Ltd., Thailand; ^d^ GABA International AG, Therwil, Switzerland; ^e^ 3 M ESPE, Minnesota, US

^*^pH measurement of the slurries (1 part of the product with 3 parts of deionized water; w/w)

^**^pH was measured in solution

#### Experimental procedure

**Fig. 2:**
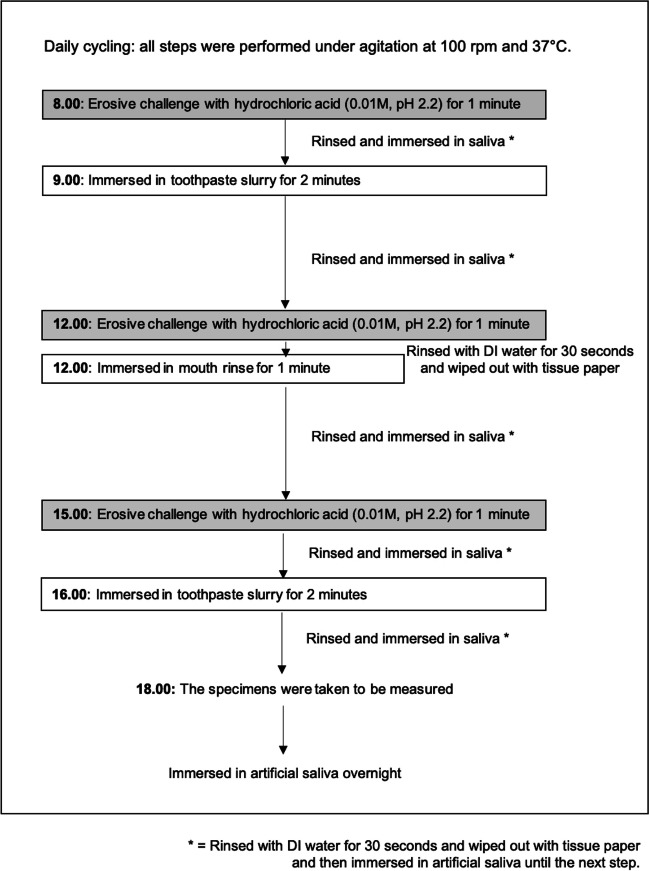
Flowchart of erosive cycling

The experiment was a cyclic procedure over a 5-day period. To form the pellicle, all specimens were immersed in artificial saliva (1.45 mM Ca; 5.4 mM PO4; 0.1 M Tris buffer; and 2.2 g/L porcine gastric mucin, pH 7.0) [38, 39] for 18 h before the onset of the experimental procedure. Each day, enamel specimens were subjected to three erosive hydrochloric acid challenges that simulated gastric reflux episodes. The specimens were immersed in 0.01 M hydrochloric acid solution (5 ml per sample) for 1 min each time. During the treatment phase, the specimens received three daily treatments that mimicked the routine of brushing twice a day and rinsing once, separately. For brushing, the specimens were immersed in toothpaste slurries for 2 min. The toothpaste slurries were prepared by mixing 1 part of the toothpaste with 3 parts of deionized water by weight. For rinsing, the specimens were immersed in mouth rinse for 1 min once a day. To avoid a potential effect associated with an abrasive process of tooth brushing, the specimen was treated with toothpaste in a slurry form without any brushing force applied. The pH of the mouth rinse and toothpaste slurries was measured using a pH electrode. Between each immersion, the specimens were washed in DI water (pH = 6.9) for 30 s. At the end of each experimental day, the specimen's surface was assessed and immersed in artificial saliva overnight. All procedures were performed under agitation at 100 rpm and 37 °C. Figure 2 summarizes the study design.

#### Measurement of enamel surface microhardness

To ensure blindness of the study, each block was assigned a random number, and the person who analyzed the blocks was unaware of the treatment. After each experimental day, five new indentations (50-g load for 5 s) were made 100 µm apart from the previous indentations. The surface hardness loss was calculated for each day using the following equation:

$$\mathrm{surface\;hardness\;loss }=\mathrm{\;surface\;hardness\;baseline}-\mathrm{surface\;hardness\;after\;treatment}$$

#### Measurement of enamel surface loss

At the end of the first, third, and fifth experimental days, the UPVC tape was carefully removed. There were 2 marker points on the block that were aligned vertically above and below the specimens. This allowed for replacement of the protective film in the exact same position. This process was carried out using a microscope with 4X magnification*.* The slabs were then scanned with a non-contact profilometer (Infinite Focus SL, Alicona, Austria). The profilometry evaluation was done linearly, and the measurement started from the sound enamel to the eroded area and then to the sound enamel on the other side.

Comparing the experimental area to the reference area on both sides indicated enamel surface loss (µm). The enamel surface loss (µm) for each slab was determined by averaging three measurements.

#### Surface topography and elemental analysis

At the end of the experimental period, 3 samples from each group were randomly selected. The stannous (Sn) and fluoride (F) contents in weight percent (wt%) on the sample surface were determined using X-ray energy dispersive spectroscopy (EDS) (Quanta250, FEI, USA). After the EDS analysis, the samples were then coated with gold. The samples were analyzed in a scanning electron microscope (SEM) (Quanta250, FEI, USA). SEM images of enamel surfaces were taken at 3000x, 5000x, and 10,000 × magnifications.

#### Statistical analysis

The mean and standard deviation (SD) of surface hardness loss and enamel surface loss was calculated. Shapiro–Wilk test and Levene’s test were performed to check the assumption of normal distribution and the equality of variances of the data, respectively. Two-way mixed ANOVA was performed to analyze the influence of treatment and the number of experimental days. A one-way ANOVA with repeated measures was conducted in all groups to compare the data among experimental days within each treatment group. The data in groups 1, 2, and 7 were not normally distributed, and therefore the Friedman test was employed for these groups.

To compare microhardness and surface loss among the different treatment groups, a one-way ANOVA with the Least Significant Difference (LSD) test was performed. SPSS Statistic (version 28) was used to analyze all the data, and the level of significance was set to 0.05.

## Results

### The effects of 1000 ppm stannous fluoride toothpaste combined with stannous mouth rinses on enamel erosion

**Fig. 3:**
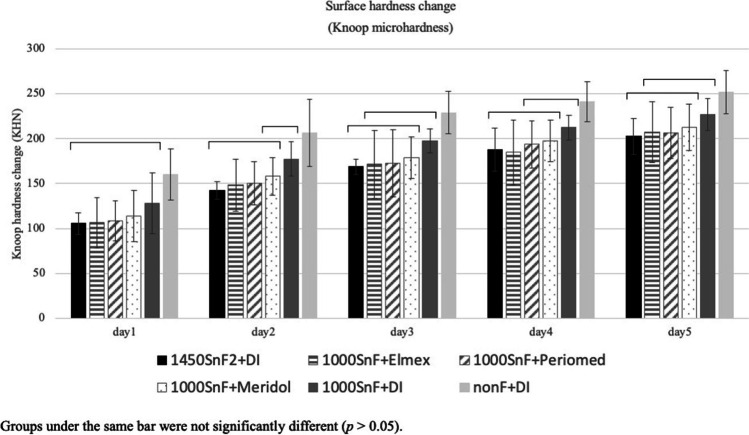
Knoop microhardness change in 1000SnF_2_ toothpaste groups and 1450SnF_2_ toothpaste alone, after 5 days of erosive cycling

For both Knoop microhardness data and profilometric analysis, two-way mixed ANOVA revealed a significant difference among the tested groups, as well as the duration of erosive challenges represented by the number of experimental days. However, their interaction was not significant. Surface microhardness change demonstrated that the 1000SnF_2_ combined with mouth rinses and the 1000SnF_2_ alone did not significantly differ from each other but were all significantly different from the negative control group (Fig. 3).

**Fig. 4:**
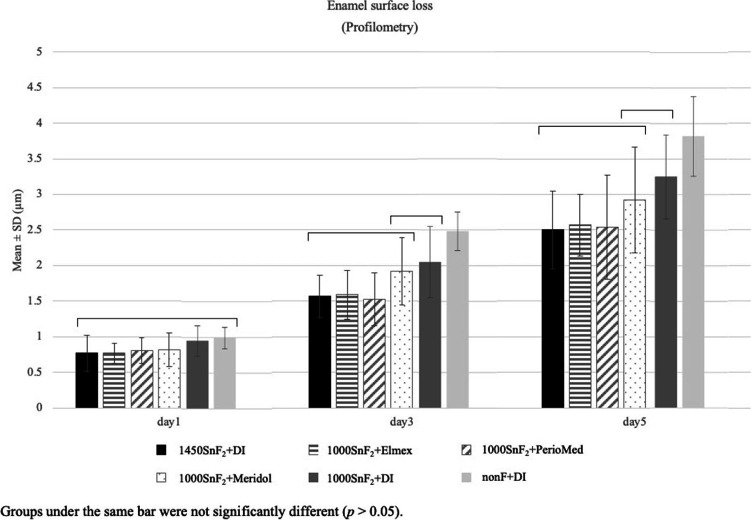
Enamel surface loss (µm) in 1000SnF_2_ toothpaste groups and 1450SnF_2_ toothpaste alone, after 5 days of erosive cycling

Profilometry data revealed that the 1000SnF_2_ + Elmex® and 1000SnF_2_ + PerioMed™ groups provided more protection against enamel surface erosion than the 1000SnF_2_ group (*p 0.05*). However, 1000SnF_2_ combined with mouth rinses had no more significant protective effect against enamel surface loss, when compared to 1450SnF_2_ alone (Fig. 4).

### The effect of 1450 ppm stannous fluoridated toothpaste combined with stannous mouth rinses on enamel erosion

**Fig. 5:**
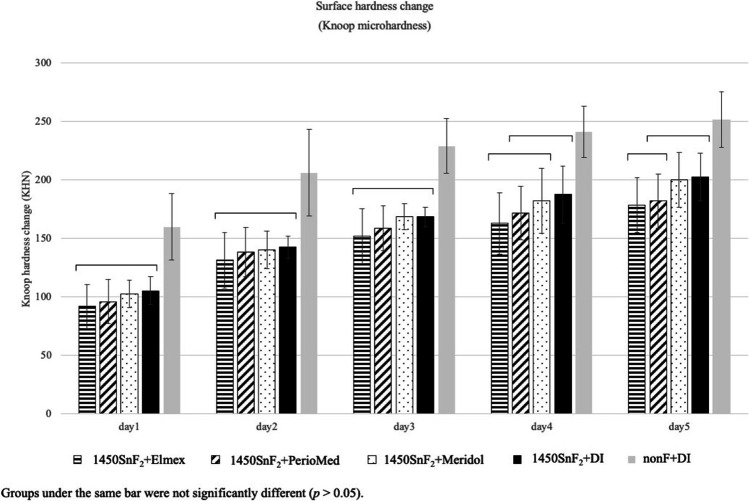
Knoop microhardness change in 1450SnF_2_ toothpaste groups, after 5 days of erosive cycling

**Fig. 6:**
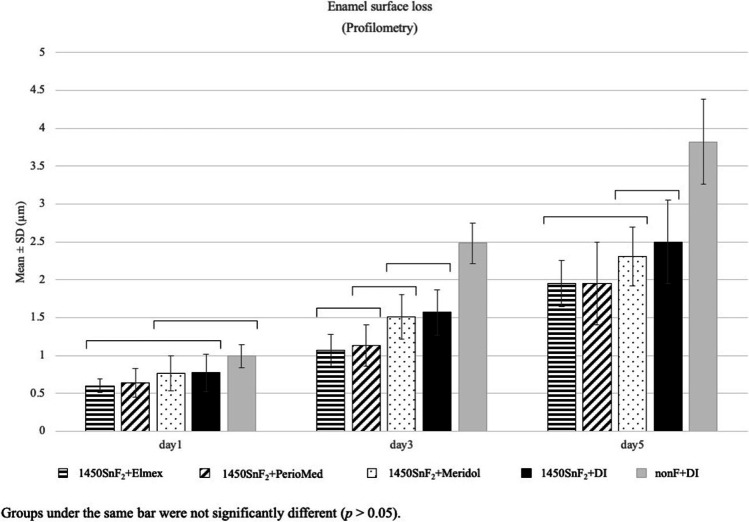
Enamel surface loss (µm) in 1450SnF_2_ toothpaste groups, after 5 days of erosive cycling

After 5 days of cycling, 1450SnF_2_ + Elmex® had the lowest microhardness change and was the only group that performed significantly better than the 1450SnF_2_ + DI group (Fig. 5). According to profilometry data, the three mouth rinse groups showed no significant difference in protection against enamel surface loss. Only the 1450SnF_2_ + elmex® and 1450SnF_2_ + periomed™, however, were significantly more effective in preventing enamel surface loss than 1450SnF2 alone (*p* < 0.05). In addition, 1450SnF_2_ + Elmex® showed less enamel surface loss than the 1450SnF_2_ + DI group on days 3 and 5. (Fig. 6).

### SEM and EDS analysis of the eroded enamel surfaces

**Fig. 7:**
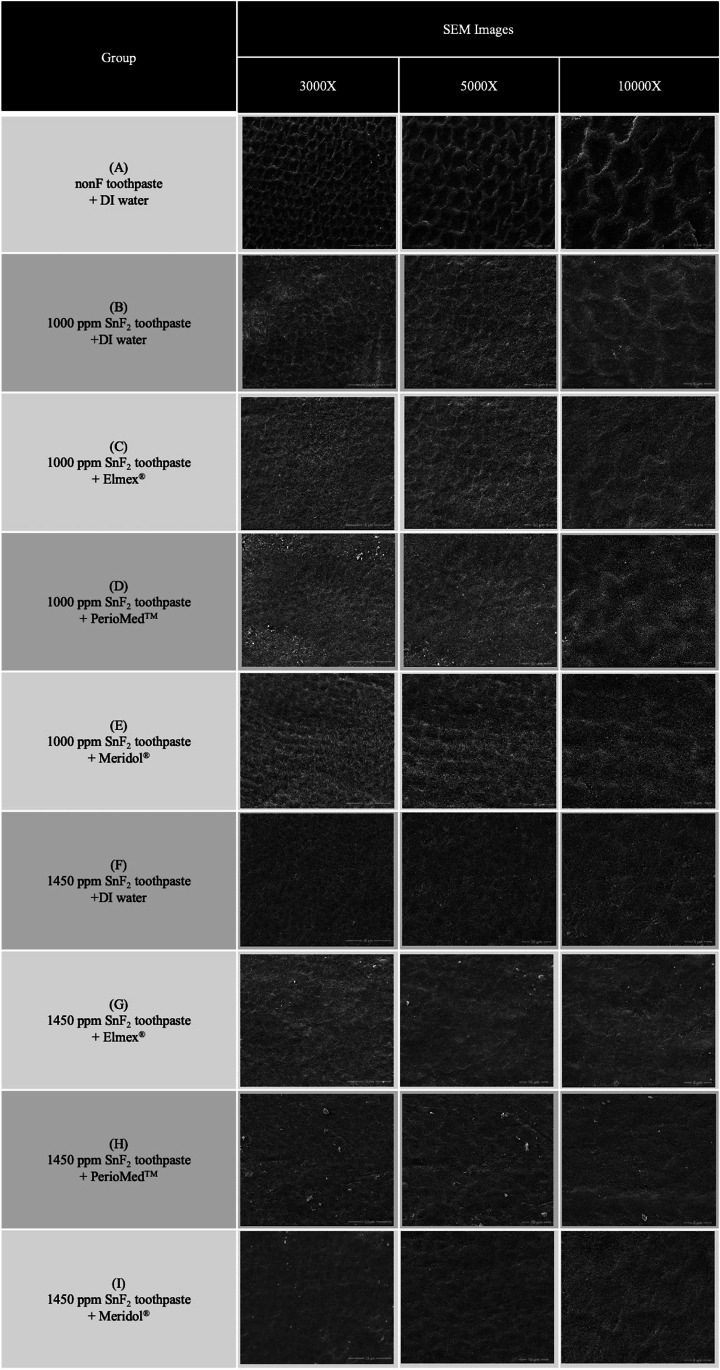
Representative scanning electron micrographs of the enamel samples collected after completion of the experiment. (**A**): nonF toothpaste + DI water group; (**B**): 1000 ppm SnF_2_ toothpaste + DI water group; (**C**): 1000 ppm SnF_2_ toothpaste + Elmex® group; (**D**): 1000 ppm SnF_2_ toothpaste + PerioMed™ group; (**E**): 1000 ppm SnF_2_ toothpaste + Meridol® group; (**F**): 1450 ppm SnF_2_ toothpaste + DI water group; (**G**): 1450 ppm SnF_2_ toothpaste + Elmer® group; (**H**): 1450 ppm SnF_2_ toothpaste + PerioMed™ group; (**I**): 1000 ppm SnF_2_ toothpaste + Meridol® group

SEM images from each group showed different degrees of enamel erosion. The enamel surface of the negative control group (Fig. 7A) exhibited a distinctive etching pattern with honeycomb-like surface topography. The 1000SnF_2_ group (Fig. 7B) also had a honeycomb surface topography, but the enamel was less eroded. The surface topography of the 1000SnF_2_ toothpaste combined with mouth rinses (Figs. 7C-7E) revealed a similar level of enamel erosion. The etching pattern was rarely seen on the surface of 1450SnF_2_ groups, with or without mouth rinse (Figs. 7F-7I). A distinct continuous surface was seen on specimens treated with 1450SnF_2_ + Elmex® and 1450SnF_2_ PerioMed™ (Figs. 7G-7H). According to EDS analysis, Sn was rarely detected on EDS samples of SnF_2_ toothpaste alone. Even though the sample treated with stannous mouth rinses deposited higher Sn in all groups, only toothpaste with PerioMed™ demonstrated a statistically significant difference. Detailed results for all groups are shown in Table 2.

**Table 2:** Elemental content (%wt) on surfaces after 5 days of the experiment

	Elemental content (%wt) mean ± s.d			
Group (*n* = 3)	Sn	F	Ca	P
Non F toothpaste	0.00 ± 0.0 ^a^	0.00 ± 0.0^a^	68.88 ± 0.3	31.12 ± 0.3
1000SnF_2_ + DI	0.04 ± 0.0 ^a,c^	1.44 ± 0.3^b^	67.67 ± 0.1	30.86 ± 0.9
1000SnF_2_ + Elmex®	1.32 ± 0.5 ^a,c^	1.52 ± 0.7^b^	68.22 ± 2.7	28.90 ± 2.3
1000SnF_2_ + Periomed™	3.60 ± 1.6 ^b^	1.47 ± 0.2^b^	65.03 ± 2.0	29.08 ± 1.2
1000SnF_2_ + Meridol®	0.77 ± 0.3 ^a,c^	1.44 ± 0.2^b^	67.81 ± 0.7	29.99 ± 0.4
1450SnF_2_ + DI	0.05 ± 0.1 ^a,c^	1.48 ± 0.3^b^	68.22 ± 1.2	30.25 ± 1.0
1450SnF_2_ + Elmex®	1.44 ± 0.3 ^c^	1.63 ± 0.3^b^	66.49 ± 0.6	30.53 ± 0.3
1450SnF_2_ + Periomed™	3.94 ± 1.8 ^b^	1.30 ± 0.4^b^	65.03 ± 1.9	29.64 ± 0.6
1450SnF_2_ + Meridol®	1.27 ± 0.5 ^a,c^	1.54 ± 0.4^b^	67.73 ± 2.6	29.46 ± 2.3

Different letters denote statistically significant differences among the groups (*p* < 0.05)

## Discussion

In the present study, we found that twice-daily treatment of human enamel specimens with stannous fluoride toothpaste and once a day with stannous fluoride mouth rinses provided an apparent protective effect against repeated hydrochloric acid erosion. Although a slightly additional protective effect was shown when mouth rinse was used together with toothpaste twice daily, it should be kept in mind that the toothpaste used in this study was stannous fluoride-containing toothpaste which has an anti-erosion effect as well. This is unlike toothpaste containing sodium fluoride or sodium monofluorophosphate, which is more widely used but less effective in preventing erosion than toothpaste containing stannous fluoride.

Interestingly, our data indicate that 1000SnF_2_ toothpaste combined with stannous mouth rinse exhibited a similar anti-erosion against HCl as 1450SnF_2_ toothpaste alone. This result could be used to recommend brushing with 1450SnF_2_ toothpaste twice daily without the use of a mouth rinse for individuals such as children or adults who cannot control their swallowing, for example. If the patient uses a 1000SnF_2_ toothpaste or if a 1450SnF_2_ toothpaste is unavailable, the additional use of stannous-containing mouth rinse once a day may be appropriate for these conditions. It is worth noting that including mouth rinse as part of oral hygiene practice involves extra costs and an additional step in the daily routine. Therefore, its use may not be necessary for everyone. It should be recommended for patients with severe conditions or those at high risk for dental erosion.

Although all stannous-treated samples in our study demonstrated a preventative effect on enamel erosion, the EDS detected the rare absence of stannous ion release in samples treated with both stannous fluoride toothpaste. These can be attributed to the complex excipient compositions in toothpaste, such as stabilizers, thickeners, detergents, and abrasives such as silica, by which the stannous ion can be adsorbed, reducing the availability of stannous ions, as opposed to stannous mouth rinses, which do not contain silica and could act as a large reservoir of stannous ions [40–43]. In addition, it should be noted that the groups receiving mouth rinses experienced greater exposure to stannous compounds. Therefore, the frequency of daily application appears to be a crucial factor in achieving the desired outcome[20, 44]. The findings suggest that a more intensive regimen could be more effective in addressing erosive challenges.

The mouth rinses used in this study have two stannous compounds, which were SnF_2_ (PerioMed™, Meridol®) and SnCl_2_ (Elmex®). Although mouth rinse contains different stannous compounds, there was still efficacy in erosion prevention [45–47]. The efficacy of stannous fluoride solutions against enamel erosion, either alone or combined with other fluoride solutions such as AmF and NaF, has been proven in several studies [26, 48–50]. Stannous chloride is also beneficial in reducing tooth erosion [21, 51], especially when combined with other fluorides (AmF or NaF), which demonstrated a more anti-erosive effect than the used SnCl_2_ solution alone [21]. The use of stannous chloride as the stannous source and sodium/amine fluoride as the fluoride source allowed the efficacy of the solution to be optimized by arranging the most effective ratio between fluoride and tin, independent of the other components [52].

Our findings demonstrated that, when used in combination with 1450 SnF_2_ toothpaste, Elmex®, and PerioMed™ had a promising preventive effect compared to Meridol®. The difference in anti-erosion efficacy of stannous-containing mouth rinses was probably related to a variety of factors, including mouth rinse pH [21], stannous compound efficacy[21], stannous and fluoride concentration [25], and the Sn/F ratio [53].

Regarding concentrations of stannous and fluoride, an in vitro study investigated the effect of various stannous concentrations, but a constant fluoride concentration, on enamel loss revealed a dose–response relationship between the stannous concentration and the protection against enamel loss [25]. A similar outcome was confirmed by an in-situ trial [54]. This could explain why Meridol®, the solution with the lowest concentration of Sn^2+^, provides the least erosion prevention in our study. Elmex® and PerioMed™ solutions with higher Sn^2+^ concentrations provided more protection. Even though Elmex® had approximately half the fluoride concentration of PerioMed™, no significant difference was found in erosion protection between the two groups. This suggests that rather than fluoride ion concentrations, the difference in erosive prevention between Elmex®, PerioMed™, and Meridol® may be due to variations in stannous ion concentrations.

From EDS analysis, PerioMed™ showed a higher amount of Sn deposited on the sample surface than other groups. However, there was no distinct difference in their ability to protect surface microhardness and enamel loss. This could imply that, whereas tin deposition is relatively resistant to acids, the effectiveness of stannous-containing products in preventing enamel erosion is not solely due to the surface deposition of Sn. Recent reports suggest that the concentration of stannous solution can affect the amount of stannous incorporated into the enamel beneath the surface [52]. Furthermore, the reaction between stannous and hydroxyapatite can result in different stannous compounds being formed [55], which may affect the efficacy of stannous products. Therefore, further research is necessary to fully understand this point.

According to our findings, the 1450 SnF_2_ toothpaste combined with either Elmex® or PerioMed™ mouth rinse demonstrated the strongest anti-erosion effect. If our findings are confirmed in a clinical trial, using stannous fluoride toothpaste (1450 ppm) twice a day, in combination with Elmex® or PerioMed™ once a day, should be advised for adults and children above the age of 12 years at high risk of tooth erosion. Although Meridol®, with less stannous ions, has less anti-erosion effect than Elmex® and PerioMed™, it is probably a suitable choice for children over 6 years old or those who are sensitive to the astringent properties of stannous. However, according to the manufacturer's instructions, Elmex® and Meridol® can be used immediately, while PerioMed™ must be mixed prior to use. So, this factor may influence a patient's compliance.

The limitation of this in vitro study is that we did not use mouth rinse exactly according to the manufacturer’s instructions (rinse 10 ml. for 30 s for Elmex ® and Meridol ®; rinse approximately 15 ml. for 1 min twice times for PerioMed™). The outcome of this in vitro study is based on a one-minute mouth rinse duration for all mouth rinses. Moreover, the result must be interpreted with caution because the dissolution behavior of the surface might be influenced by the presence of pellicles and saliva under in situ*/ *in vivo conditions. We used polished enamel to achieve a flat surface, which is needed for reliable Knoop surface hardness and profilometry testing. The surface layer, which contains a higher concentration of fluoride and phosphate, was removed, and is more prone to erosion than natural enamel. Therefore, this in vitro study may reveal a more aggressive scenario than occurring in the in vivo situation. Future research should examine the interaction of these products with biological factors, particularly saliva, by conducting an in-situ study or clinical trial.

## Conclusion

In conclusion, under the used laboratory conditions, no statistically significant difference was found between the three mouth rinses. However, Elmex® and PerioMed™ provided better enamel protection against erosion than Meridol®. Although the stannous fluoride toothpaste (either 1450SnF_2_ or 1000SnF_2_) alone can reduce enamel erosion, the additional use of stannous mouth rinse proved to be a more effective anti-erosion measure.

## Data Availability

The data that support the findings of this study are available from the corresponding author upon reasonable request.

## References

[1] Schlueter N, Amaechi BT, Bartlett D, Buzalaf MAR, Carvalho TS, Ganss C, Hara AT, Huysmans M, Lussi A, Moazzez R, Vieira AR, West NX, Wiegand A, Young A, Lippert F (2020). Terminology of Erosive Tooth Wear: Consensus Report of a Workshop Organized by the ORCA and the Cariology Research Group of the IADR. Caries Res.

[2] Wang GR, Zhang H, Wang ZG, Jiang GS, Guo CH (2010). Relationship between dental erosion and respiratory symptoms in patients with gastro-oesophageal reflux disease. J Dent.

[3] Moazzez R, Bartlett D (2014). Intrinsic causes of erosion. Monogr Oral Sci.

[4] Scaramucci T, Carvalho JC, Hara AT, Zero DT (2015) Causes of dental erosion: intrinsic factors. In: Amaechi B. (ed) Dental erosion and its clinical management. Springer, Cham, pp. 35–67. 10.1007/978-3-319-13993-7_3

[5] da Silva CS, Epifanio M, Scheeffer VA, Melere MU, Steinhaus C (2021). High prevalence of dental erosion in children with erosive esophagitis. Ann Pediatr Child Health.

[6] Kotsanos N, Birkhed D, Kotsanos N, Sarnat H, Park K (2022). Tooth Wear in Children and Adolescents. Pediatric Dentistry.

[7] Li Y, Wang Z, Fang M, Tay FR, Chen X (2022). Association between gastro-oesophageal reflux disease and dental erosion in children: A systematic review and meta-analysis. J Dent.

[8] Pace F, Pallotta S, Tonini M, Vakil N, Bianchi Porro G (2008). Systematic review: gastro-oesophageal reflux disease and dental lesions. Aliment Pharmacol Ther.

[9] Picos A, Badea ME, Dumitrascu DL (2018). Dental erosion in gastro-esophageal reflux disease. A systematic review. Clujul Med.

[10] Kanzow P, Wegehaupt FJ, Attin T, Wiegand A (2016). Etiology and pathogenesis of dental erosion. Quintessence Int.

[11] Ganss C, Lussi A, Schlueter N (2014). The histological features and physical properties of eroded dental hard tissues. Monogr Oral Sci.

[12] Lussi A, Carvalho TS (2015). The future of fluorides and other protective agents in erosion prevention. Caries Res.

[13] Faller RV, Noble WH (2018). Protection From Dental Erosion: All Fluorides are Not Equal. Compend Contin Educ Dent.

[14] Chan AS, Tran TTK, Hsu YH, Liu SYS, Kroon J (2020). A systematic review of dietary acids and habits on dental erosion in adolescents. Int J Paediatr Dent.

[15] Schlueter N, Luka B (2018). Erosive tooth wear - a review on global prevalence and on its prevalence in risk groups. Br Dent J.

[16] Patel A, Amaechi BT, Brady C (2015) Prevention and control of dental erosion: gastroesophageal reflux disease management. In: Amaechi B. (ed) Dental erosion and its clinical management. Springer, Cham, pp. 203–224. 10.1007/978-3-319-13993-7_12

[17] Johansson A-K, Arnadottir I, Koch G, Poulsen S, Koch G, Poulsen S, Espelid I, Haubek D (2017). Dental erosion. Pediatric Dentistry: A Clinical Approach.

[18] Buzalaf MAR, De Almeida Baldini Cardoso C, Magalhães AC, Amaechi BT (2015) Prevention and control of dental erosion: patient self-care. In: Amaechi B. (ed) Dental erosion and its clinical management. Springer International Publishing, pp. 133–150. 10.1007/978-3-319-13993-7_8

[19] World Health Organization (2023) Oral Health, https://www.who.int/news-room/fact-sheets/detail/oral-health. Accessed 20 June 2023

[20] da Silva CV, Nazello JL, de Freitas PM (2017). Frequency of Application of AmF/NaF/SnCl2 Solution and Its Potential in Inhibiting the Progression of Erosion in Human Dental Enamel - An In Vitro Study. Oral Health Prev Dent.

[21] Ganss C, Schlueter N, Hardt M, Schattenberg P, Klimek J (2008). Effect of fluoride compounds on enamel erosion in vitro: a comparison of amine, sodium and stannous fluoride. Caries Res.

[22] Attin T, Becker K, Wiedemeier DB, Schmidlin PR, Wegehaupt FJ (2017). Anti-erosive effect of a self-assembling peptide gel. Swiss Dent J.

[23] Schlueter N, Neutard L, von Hinckeldey J, Klimek J, Ganss C (2010). Tin and fluoride as anti-erosive agents in enamel and dentine in vitro. Acta Odontol Scand.

[24] Körner P, Nguyen TP, Hamza B, Attin T, Wegehaupt FJ (2021). Enamel Softening Can Be Reduced by Rinsing with a Fluoride Mouthwash Before Dental Erosion but Not with a Calcium Solution. Oral Health Prev Dent.

[25] Schlueter N, Klimek J, Ganss C (2009). In vitro efficacy of experimental tin- and fluoride-containing mouth rinses as anti-erosive agents in enamel. J Dent.

[26] Huysmans MC, Young A, Ganss C (2014). The role of fluoride in erosion therapy. Monogr Oral Sci.

[27] Magalhães AC, Wiegand A, Rios D, Buzalaf MAR, Lussi A (2011). Fluoride in dental erosion. Monogr Oral Sci.

[28] Moser C, Baumann T, Lussi A, Carvalho TS (2021). Is the Erosion-Protective Effect Still Maintained when Tin Concentrations Are Reduced in Mouth Rinse Solutions?. Caries Res.

[29] Rakhmatullina E, Beyeler B, Lussi A (2013). Inhibition of enamel erosion by stannous fluoride containing rinsing solutions. Schweiz Monatsschr Zahnmed.

[30] de Souza BM, Santi LRP, de Souza SM, Buzalaf MAR, Magalhães AC (2018). Effect of an experimental mouth rinse containing NaF and TiF(4) on tooth erosion and abrasion in situ. J Dent.

[31] O'Toole S, Mistry M, Mutahar M, Moazzez R, Bartlett D (2015). Sequence of stannous and sodium fluoride solutions to prevent enamel erosion. J Dent.

[32] O'Toole S, Bartlett DW, Moazzez R (2016). Efficacy of sodium and stannous fluoride mouthrinses when used before single and multiple erosive challenges. Aust Dent J.

[33] Wiegand A, Gutsche M, Attin T (2007). Effect of olive oil and an olive-oil-containing fluoridated mouthrinse on enamel and dentin erosion in vitro. Acta Odontol Scand.

[34] Souza BM, Lima LL, Comar LP, Buzalaf MA, Magalhães AC (2014). Effect of experimental mouthrinses containing the combination of NaF and TiF4 on enamel erosive wear in vitro. Arch Oral Biol.

[35] Castilho ARFd, Salomão PMA, Buzalaf MAR, Magalhães AC (2015). Protective effect of experimental mouthrinses containing NaF and TiF4 on dentin erosive loss in vitro. J Appl Oral Sci.

[36] Ramos-Oliveira TM, Silva CV, Nunes PM, Turssi CP, Rechmann P, Freitas PM (2017). AmF/NaF/SnCl2 solution reduces in situ enamel erosion - profilometry and cross-sectional nanoindentation analysis. Braz Oral Res.

[37] Passos VF, Rodrigues LKA, Santiago SL (2018). The effect of magnesium hydroxide-containing dentifrice using an extrinsic and intrinsic erosion cycling model. Arch Oral Biol.

[38] Turssi CP, Amaral FLB, França FMG, Basting RT, Hara AT (2019). Effect of sucralfate against hydrochloric acid-induced dental erosion. Clin Oral Investig.

[39] Passos VF, de Vasconcellos AA, Pequeno JH, Rodrigues LK, Santiago SL (2015). Effect of commercial fluoride dentifrices against hydrochloric acid in an erosion-abrasion model. Clin Oral Investig.

[40] Ganss C, von Hinckeldey J, Tolle A, Schulze K, Klimek J, Schlueter N (2012). Efficacy of the stannous ion and a biopolymer in toothpastes on enamel erosion/abrasion. J Dent.

[41] Ganss C, Möllers M, Schlueter N (2017). Do Abrasives Play a Role in Toothpaste Efficacy against Erosion/Abrasion?. Caries Res.

[42] Ionta FQ, Dos Santos NM, Mesquita IM, Dionísio EJ, Cruvinel T, Honório HM, Rios D (2019). Is the dentifrice containing calcium silicate, sodium phosphate, and fluoride able to protect enamel against chemical mechanical wear? An in situ/ex vivo study. Clin Oral Investig.

[43] Lucchese A, Bertacci A, Lo Giudice A, Polizzi E, Gherlone E, Manuelli M, Chersoni S, Moro D, Valdrè G (2020) Stannous fluoride preventive effect on enamel erosion: An In Vitro Study. J Clin Med 9. 10.3390/jcm9092755 10.3390/jcm9092755 PMC7563875 32858829

[44] Khambe D, Eversole SL, Mills T, Faller RV (2014). Protective effects of SnF2 - Part II. Deposition and retention on pellicle-coated enamel. Int Dent J.

[45] Ganss C, Neutard L, von Hinckeldey J, Klimek J, Schlueter N (2010). Efficacy of a tin/fluoride rinse: a randomized in situ trial on erosion. J Dent Res.

[46] Kensche A, Kirsch J, Mintert S, Enders F, Pötschke S, Basche S, König B, Hannig C, Hannig M (2017). Impact of customary fluoride rinsing solutions on the pellicle’s protective properties and bioadhesion in situ. Sci Rep.

[47] Schlueter N, Klimek J, Ganss C (2011). Efficacy of tin-containing solutions on erosive mineral loss in enamel and dentine in situ. Clin Oral Investig.

[48] Ganss C, Lussi A, Sommer N, Klimek J, Schlueter N (2010). Efficacy of fluoride compounds and stannous chloride as erosion inhibitors in dentine. Caries Res.

[49] Algarni AA, Lippert F, Hara AT (2015) Efficacy of stannous, fluoride and their their combination in dentin erosion prevention in vitro. Braz Oral Res 29. 10.1590/1807-3107BOR-2015.vol29.0081 10.1590/1807-3107BOR-2015.vol29.0081 26106907

[50] Algarni AA, Mussi MC, Moffa EB, Lippert F, Zero DT, Siqueira WL, Hara AT (2015). The impact of stannous, fluoride ions and its combination on enamel pellicle proteome and dental erosion prevention. PLoS One.

[51] Ellingsen JE (1986). Scanning electron microscope and electron microprobe study of reactions of stannous fluoride and stannous chloride with dental enamel. Scand J Dent Res.

[52] Schlueter N, Hardt M, Lussi A, Engelmann F, Klimek J, Ganss C (2009). Tin-containing fluoride solutions as anti-erosive agents in enamel: an in vitro tin-uptake, tissue-loss, and scanning electron micrograph study. Eur J Oral Sci.

[53] Schlueter N, Klimek J, Ganss C (2009). Effect of stannous and fluoride concentration in a mouth rinse on erosive tissue loss in enamel in vitro. Arch Oral Biol.

[54] Schlueter N, Klimek J, Ganss C (2009). Efficacy of an experimental tin-F-containing solution in erosive tissue loss in enamel and dentine in situ. Caries Res.

[55] Babcock FD, King JC, Jordan TH (1978). The reaction of stannous fluoride and hydroxyapatite. J Dent Res.

